# Can oral health and oral‐derived biospecimens predict progression of dementia?

**DOI:** 10.1111/odi.13201

**Published:** 2020-01-06

**Authors:** Miranda E. Orr, Kelly R. Reveles, Chih‐Ko Yeh, Eric H. Young, Xianlin Han

**Affiliations:** ^1^ Barshop Institute for Longevity and Aging Studies UT Health San Antonio San Antonio Texas; ^2^ Geriatric Research, Education & Clinical Center and Research Service South Texas Veterans Health Care System San Antonio Texas; ^3^ Department of Medicine UT Health San Antonio San Antonio Texas; ^4^ Biggs Institute for Alzheimer’s and Neurodegenerative Diseases UT Health San Antonio San Antonio Texas; ^5^ Gerontology and Geriatric Medicine Sticht Center for Healthy Aging and Alzheimer's Prevention Wake Forest School of Medicine Winston‐Salem North Carolina; ^6^ College of Pharmacy The University of Texas at Austin Austin Texas; ^7^ Pharmacotherapy Education & Research Center UT Health San Antonio San Antonio Texas; ^8^ Comprehensive Dentistry School of Dentistry UT Health San Antonio San Antonio Texas

**Keywords:** Alzheimer's disease, dementia, oral health, oral microbiome, saliva lipidomics, volatile organic compounds

## Abstract

Growing evidence indicates that oral health and brain health are interconnected. Declining cognition and dementia coincide with lack of self‐preservation, including oral hygiene. The oral microbiota plays an important role in maintaining oral health. Emerging evidence suggests a link between oral dysbiosis and cognitive decline in patients with Alzheimer's disease. This review showcases the recent advances connecting oral health and cognitive function during aging and the potential utility of oral‐derived biospecimens to inform on brain health. Collectively, experimental findings indicate that the connection between oral health and cognition cannot be underestimated; moreover, oral biospecimens are abundant and readily obtainable without invasive procedures, which may help inform on cognitive health.

## 1 INTRODUCTION

**Figure 1:**
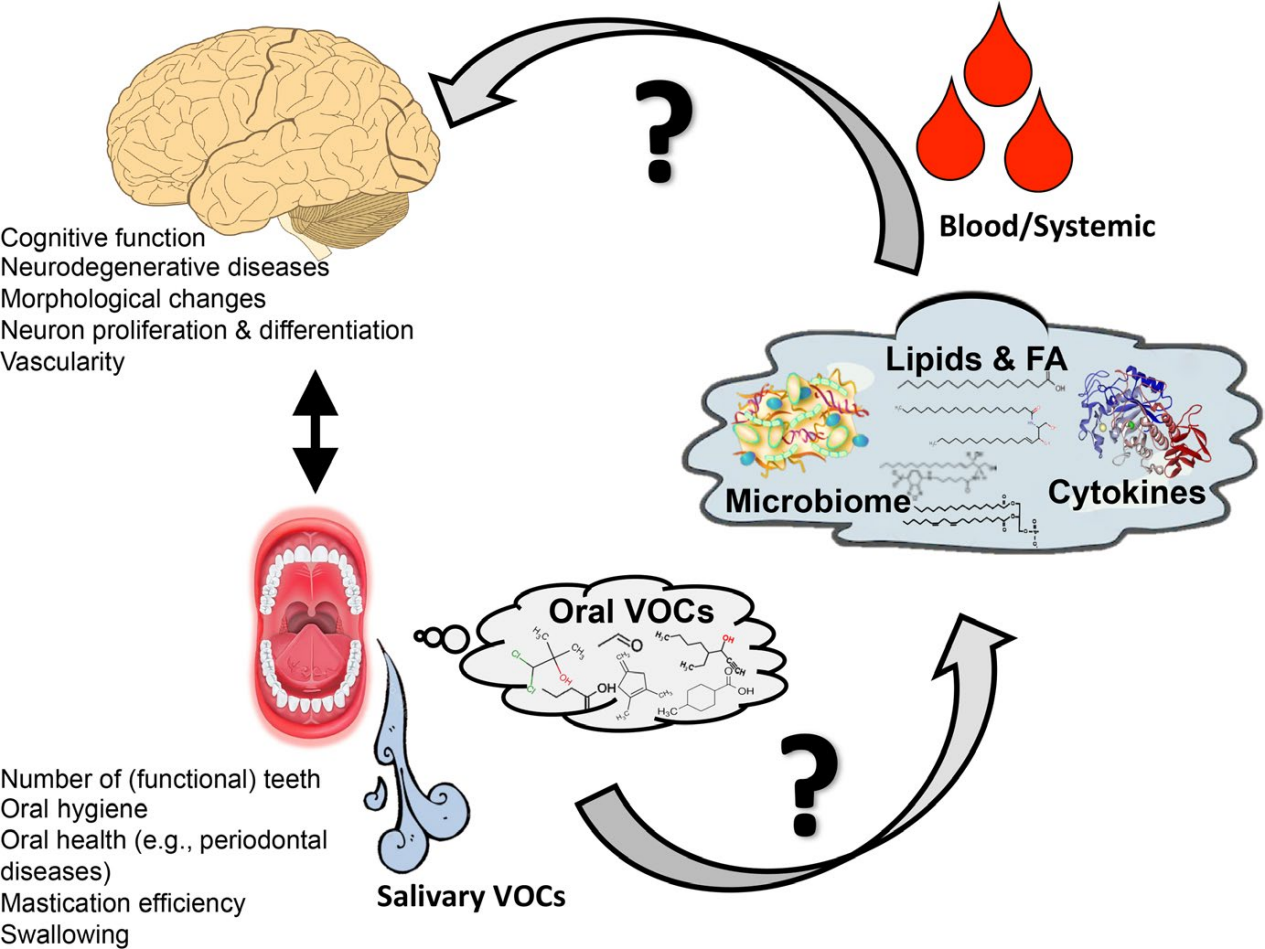
Schematic interaction of oral health and brain cognitive function and possible mechanisms linking them. FA, fatty acids; VOC, volatile organic compounds

Alzheimer's disease (AD) is a chronic neurodegenerative disorder and the leading cause of dementia worldwide. AD affects more than 5.7 million Americans, predominately in those 65 years and older (Alzheimer's Association Report, 2018; Hebert, Weuve, Scherr, & Evans, 2013). Clinically, AD is diagnosed by a progressive decline in cognition and memory resulting in an inability to function independently in everyday life. A confirmed AD diagnosis requires the presence of amyloid beta (Aβ) plaques and tau‐containing neurofibrillary tangles (NFTs) in brain tissue analyzed postmortem. Advances in brain imaging and cerebral spinal fluid measurements have greatly aided in accurate AD diagnoses that closely parallel postmortem histopathology (Johnson, Fox, Sperling, & Klunk, 2012). Historically, therapeutic interventions to treat AD have focused on targeting brain pathologies (primarily Aβ plaques, and more recently tau). Due to the limited success in developing disease‐modifying treatments with this strategy, a growing appreciation for systemic health and its role in AD pathogenesis is emerging. Indeed, AD patients suffer more comorbid health conditions than age‐matched older adults, including diabetes mellitus, osteoporosis, and depression (Wang, Wu, Tee, & Lo, 2018). Among these, recent preclinical and epidemiological studies have suggested an interconnection between oral health and brain health. Here, we will review the potential role of the oral cavity and oral‐derived biomarkers for AD diagnosis and tracking disease progression (Figure 1). Table 1 provides an overview of studies evaluated.

**Table 1:** Overview of studies evaluating the association between oral health and cognition

Study	Study type	Population	Outcome(s)	Results
Luo et al., 2015	Human cohort study	Dementia (*n* = 120) MCI (*n* = 554) Cognitive normal (*n* = 2,389)	Mean (*SD*) teeth missing	Dementia: 18.7 (11.0) MCI: 11.8 (9.9) Cognitive normal: 9.3 (9.3)
Park et al., 2013	Human cohort study	Number of teeth lost: 6–10 teeth >10 teeth	Cognitive impairment based on MMSE < 24	6–10 teeth: aOR 1.99, 95% CI 1.08–3.69 >10 teeth: aOR 2.25, 95% CI 1.26–4.02
Takeuchi et al., 2017	Human cohort study	Number of remaining teeth: ≥20 (*n* = 893) 10–19 (*n* = 328) 1–9 (*n* = 204) 0 (*n* = 121)	All‐cause dementia	10–19: aHR 1.62, 95% CI 1.06–2.46 1–9: aHR 1.81, 95% CI 2.94 0: aHR 1.63, 95% 0.95–2.80
Gatz et al., 2006	Human case–control twin study	Demented (*n* = 82) Not demented (*n* = 82)	Oral disease from tooth loss	Demented versus non‐demented: OR 3.6, 95% CI 1.34–9.70
Stein et al., 2007	Human cohort study	Number of non‐third molars: 0 (*n* = 25) 1–9 (*n* = 26) 10–16 (*n* = 27) 17–28 (*n* = 66) Number of non‐third molars with apoE4 allele: 0 (*n* = 3) 1–9 (*n* = 6) 10–16 (*n* = 5) 17–28 (*n* = 18)	Dementia	All participants 0: OR 0.9, 95% CI 0.25–3.12 1–9: OR 1.8, 95% CI 0.58–5.46 10–16: OR 0.4, 95% 0.10–1.76 ApoE4 allele: 0: OR 0.1, 95% CI 0.01–3.7 1–9: OR 0.5, 95% CI 0.04–5.6 10–16: OR 0.3, 95% CI 0.02–3.6
Shimazaki et al., 2001	Human cohort study	Dentition status: >20 teeth (*n* = 150) 1–19 teeth with dentures (*n* = 440) 1–19 teeth not using dentures (*n* = 371) Edentulous using dentures (*n* = 621) Edentulous not using dentures (*n* = 347)	Six‐year mental impairment	1–19 teeth with dentures: OR 1.9, 95% CI 0.8–4.6 1–19 teeth not using dentures: OR 2.3, 95% CI 0.9–5.8 Edentulous using dentures: OR 1.7, 95% CI 0.7–4.0 Edentulous not using dentures: OR 2.4, 95% CI 0.9–6.5
Stewart et al., 2013	Human cohort study	Quartile of oral health parameters: Q1 (*n* = 264) Q2 (*n* = 186) Q3 (*n* = 110) Q4 (*n* = 46)	Cognitive impairment	Per oral health quartile increase: OR 0.56, 95% CI 0.48–0.67
Chen et al., 2015	Human cross‐sectional study	CIND (*n* = 57) Dementia (*n* = 51) No cognitive impairment (*n* = 492)	Number of carious teeth or retained roots while adjusting for the capacity to perform oral hygiene	CIND: RR 1.66, 95% CI 1.13–2.46 Dementia: RR 1.82, 95% CI 1.23–2.70
Cho et al., 2018	Human cohort study	Normal cognitive ability (*n* = 284) Dementia (*n* = 61)	Oral health	Demented versus non‐demented: OR 2.29, 95% CI 1.08–4.83
Oue et al., 2013	Interventional, prospective study (J20 mice)	Maxillary molar teeth removed (*n* = 10) Control group with intact molars (*n* = 10)	Impact of tooth loss on acquisition (learning) versus retention (memory) latency	Retention versus acquisition latency (*p* < .05) Retention latency: 293.6 + 6.1 s Acquisition latency: 88.9 + 17.4 s
Oue et al., 2016	Interventional, prospective study (Tg2576 mice)	Maxillary molar teeth removed (*n* = 9) Control group with intact molars (*n* = 10)	Impact of tooth loss on acquisition (learning) and retention (memory) latency	Acquisition latency (*p* > .05): Molar teeth removed: 89.0 + 17.9 s Control group: 172.0 + 40.7 s Retention latency (*p* < .05): Molar teeth removed: 300.0 + 0 s Control group: 296.7 + 3.3 s
He et al., 2014	Interventional, prospective study (SAMP8 mice)	4‐month‐old mice: Alveolar nerve transection (experimental) (*n* = 20) Sham surgery (control) (*n* = 20) 7‐month‐old mice: Alveolar nerve transection (experimental) (*n* = 10) Sham surgery (control) (*n* = 10)	Escape latency Learning rate	Escape latency significantly greater in elderly experimental group than elderly control group in five‐minute acquisition session (*p* < .05) Elderly control: 39.70 + 14.84 s Elderly experimental: 63.60 + 15.31 s Learning rate in elderly mice significantly poorer in experimental group versus controls (*p* < .05) Elderly control: 18.50 + 5.44 Elderly experimental: 25.90 + 6.21
Kubo et al., 2017	Interventional, prospective study (SAMP8 mice)	Molars removed (*n* = 33) Molars intact (*n* = 33)	Plasma cortisol levels Time in Morris water maze	Higher plasma cortisone levels in early tooth loss group (*p* = .016) Early tooth loss group required more time in Morris water maze test (*p* = .016)

Abbreviations: aHR, adjusted hazard ratio; aOR, adjusted odds ratio; CIND, cognitive impairment, no dementia; MCI, mild cognitive impairment; MMSE, mini‐mental state examination; RR, relative risk; *SD*, standard deviation.

## 2 ORAL HEALTH AND COGNITIVE IMPAIRMENT—CLINICAL STUDIES

Oral health tends to deteriorate as cognitive function declines (Martande et al., 2014; Ribeiro, Costa, Ambrosano, & Garcia, 2012). Reasons for this observation are likely multifaceted; for example, individuals with dementia often are limited by resources, capabilities for oral hygiene, and receive less dental care than the general population (Teng, Lin, & Yeh, 2016). Nevertheless, dementia has been found to be a strong predictor of poor oral health including the severity of periodontitis (Rapp, Sourdet, Vellas, & Lacoste‐Ferre, 2017; Syrjala et al., 2012; Zenthofer et al., 2017; Zimmerman et al., 2017). Additionally, older adults with dementia develop multiple oral health problems related to hard tissues (e.g., coronal and root caries), periodontal tissues (e.g., gingival bleeding, periodontal pockets, and dental plaques), mucosal lesions, and lower salivary flow rates/xerostomia (Delwel et al., 2017, 2018). Some studies do not find significant associations between oral health and cognitive decline (Shimazaki et al., 2001; Stewart et al., 2013) to suggest that oral care capacity does not solely mediate the association between cognition and oral health. However, current epidemiological studies suggest a strong link between oral health/function (that may occur independent of maintaining oral care) and deterioration of cognitive health during aging (Chen, Clark, Chen, & Naorungroj, 2015; Cho et al., 2018).

Epidemiological studies have noted a bi‐directional association between oral health and dementia. While oral hygiene routines can be negatively affected by decreased cognition, oral health changes can drive cognitive decline. The number of teeth in young ages is a strong predictor of dementia, as well as other oral health issues (Luo et al., 2015; Park et al., 2013; Takeuchi et al., 2017). The relationship between the number of teeth and cognitive function has been robustly confirmed in multiple independent studies. These include a large twin study (11,884 twin pairs) designed to evaluate genetic and environmental influences on AD (Gatz et al., 2006) and the Nun Study of Aging and Alzheimer's Disease, a longitudinal study that began in 1986 to examine the onset of AD in a study cohort with similar environments and lifestyles (Stein, Desrosiers, Donegan, Yepes, & Kryscio, 2007). Similar findings have been reported globally, including The Shanghai Aging Study, a prospective cohort study of 3,000 Chinese adults aged 60 years and older. This study reported significantly fewer teeth in individuals with dementia than those with mild cognitive impairment (MCI) and normal cognitive health (18.7, 11.8, and 9.3 teeth lost, respectively) (Luo et al., 2015). The study also found that patients who had fewer teeth had an increase in colonization of periodontal bacteria. Other large clinical cohort studies have documented better cognitive function in persons with good mastication efficiency and/or more functional teeth even after adjusted for a large number of confounding factors (Miquel, Aspiras, & Day, 2018) to suggest that a reduction in tooth number may be overcome if chewing efficiency is preserved.

The specific role oral health plays in dementia pathogenesis is poorly understood and likely involves multiple etiologies. For example, poor oral health can give rise to pain, infection, and lack of gustation, all of which may alter the neural input, functional memories, and desire. Moreover, individuals with AD have poor swallowing function (Takagi et al., 2017) which also contributes to decreased ability to ingest certain foods and in turn affects the oral environment and systemic health. A recent MRI morphometry study of non‐demented dentate and edentulous subjects has shown atrophy of gray matter in the hippocampus, caudate nucleus, and temporal pole of the right hemisphere in edentulous human subjects, suggesting tooth loss increases the risks for atrophy of brain areas related to memory, learning, and cognition (Kobayashi et al., 2018). Preclinical studies in model organisms offer critical insight into mechanism mediating downstream consequences of tooth removal, and the ability to derive causation—key studies are highlighted in the following section.

## 3 ORAL HEALTH AND COGNITIVE IMPAIRMENT—MECHANISTIC INSIGHT FROM PRECLINICAL STUDIES

Changes to the oral cavity may influence health conditions of the body, and its importance to cognitive health and brain function in adults as they age has been recently recognized and reviewed (Miquel et al., 2018; Tran et al., 2018). The activity of eating involves sensory, motor, pleasure, and memory‐forming neuronal circuitry and brain regions. Mastication, or chewing, forms functional memories and becomes a basic and pleasurable physical act involving numerous brain regions through the creation of sensory inputs throughout the central nervous system via the trigeminal nuclei (Ono, Yamamoto, Kubo, & Onozuka, 2010). This elaborate process is akin to brain exercise requiring coordinated neuromuscular and somatosensory control and results in increased blood flow, blood oxygenation, and activation of numerous cortical brain regions including the hippocampus (Hasegawa et al., 2013; Hirano et al., 2013; Miyake et al., 2012; Onozuka et al., 2002), a brain region critical for learning and memory that is susceptible to AD pathogenesis*.* In addition, mastication leads to the expression of brain‐derived neurotrophic factor (BDNF), as well as its receptor, tyrosine kinase receptor B. Expression of BDNF via mastication leads to neuronal cell proliferation, differentiation, and synapse formation (Lu, 2003; Vicario‐Abejon, Owens, McKay, & Segal, 2002). Disrupted signaling between the oral cavity and the brain may interrupt important neurobiological processes that contribute to negative health outcomes, including brain function and memory. Masticatory dysfunction also leads to the downregulation of BDNF, which results in a decrease in neuronal progenitor cells and functional neurons (Smith, 2016). Experimental studies in model organisms offer an opportunity to carefully investigate this association.

Mouse models of AD pathology have been tested for the effects of tooth extraction and mastication on AD‐associated Aβ accumulation, neuron loss, and behavioral performance. Transgenic J20 mice accumulate AD‐associated Aβ protein and acquire behavioral impairments that are dependent upon age (Mucke et al., 2000). Tooth extraction of young, 6‐month‐old adult J20 transgenic mice resulted in poor memory retention, as assessed four months later, which occurred coincident with greater Aβ pathology and neuron cell loss than J20 mice with intact teeth (Oue et al., 2013). In contrast, tooth extraction performed on middle aged, 14‐month‐old Tg2576 mice, a different Aβ‐producing AD mouse model, did not alter learning and memory or alter Aβ production (Oue et al., 2016). Together, these results suggest that tooth extraction, particularly at a young age, may be a critical mediator of AD‐associated brain structural changes that influence memory formation and retention in later life.

Age‐dependent effects of trigeminal nerve damage without tooth extraction have been reported among studies using the senescence‐accelerated mouse strain P8, SAMP8, a model of accelerated aging and AD. Experimental results suggested that trigeminal nerve damage in young mice (i.e., 4‐month‐old SAMP8 mice) did not impact learning and memory at older ages (i.e., 8 or 11 months). However, nerve damage inflicted in 8‐month‐old adult SAMP8 mice caused deficits in learning and memory in 11‐month‐old mice coincident with cholinergic neuron loss in the hippocampus and basal forebrain (He et al., 2014). Interestingly, tooth extraction in very young SAMP8 mice, (1‐month‐old, an age immediately following tooth eruption) (Kubo et al., 2017) produced learning and memory deficits similar to that observed in the 8‐month‐old nerve‐damaged mice (He et al., 2014; Kondo et al., 2016). In both cohorts, the oral stress negatively impacted structure and function of the hippocampus via neuronal loss or damage. Similarly, a decrease in hippocampal‐dependent spatial learning ability was observed in SAMP8 and SAMR1 (senescence‐accelerated mouse resistant 1) mice that were fed a soft food diet (Yamamoto & Hirayama, 2001). The results from this study suggest that reduced activity from chewing soft food may result in changes in afferent impulses, which can cause alterations in neural pathways. Therefore, loss of sensory input from the teeth, and not the physical tooth loss, is critical for spatial learning and memory in rodents. Environmental enrichment (i.e., increased levels of motor, sensory, social, and cognitive stimuli) (Kondo et al., 2016) or molar restoration (Iida et al., 2014) partially restored spatial memory function and gene expression important for learning and memory, respectively, to suggest that there may be opportunities for therapeutic intervention. While the use of different mouse models may contribute to differences in findings, overall these studies indicate that tooth extraction impacts AD‐associated Aβ pathology, brain structure, and function in an age‐dependent manner. Notably, the preclinical studies to date have not addressed whether oral health stressors affect tau protein processing and/or pathogenesis similar to that described for Aβ. Tau pathology closely tracks with neurodegeneration and dementia in AD; therefore, future studies using tau transgenic mice and/or evaluating tau in the above‐mentioned AD mouse models may greatly aid the understanding of the complex connection between oral health and AD pathogenesis. While the physical and structural changes associated with tooth removal cannot be underestimated, AD is complex. In the following sections, we highlight important oral health measures beyond that of neural input and sensation that, with recent advances in technology and bioinformatics, are aiding in the understanding of the complex interplay between oral and cognitive health.

## 4 THE ORAL CAVITY AS A RICH SOURCE FOR POTENTIAL DEMENTIA‐RELATED BIOMARKERS

While the brain is an inaccessible organ, the oral cavity is a rich depot for collecting non‐invasive biospecimen data including cells, saliva, microbiota, proteins, lipids, and other metabolites found in exhaled breath (Table 2). Experimental and clinical studies suggest a possible link among these biomarkers, oral health, and cognitive decline. Here, we provide a brief review of these associations.

**Table 2:** Potential oral biomarkers for AD diagnosis

Biomarkers
Oral microbiome (e.g., presence in AD brain)
Volatile organic compounds (e.g., unique profiles among neurodegenerative diseases)
Salivary proteomics (e.g., Aβ peptides, tau, and lactoferrin, salivary acetylcholinesterase activity linked to AD)
Salivary lipidomics (a new frontier for AD)

### 4.1 Oral microbiome

The human microbiome is known to play a role in the development of AD, owing to the vast functions of the microbiome in human health. For example, the oral and gut microbiomes aid in the metabolism of short‐chain fatty acids, organic acids and vitamins, and transforming bile salts, lipids, and amino acids (Canfora, Jocken, & Blaak, 2015; Takahashi, 2015). Thus, changes in the microbiota (i.e., dysbiosis) could alter the function of the community and have a significant impact on health. Most of the prior literature has focused on the association of gut microbiome dysbiosis with AD. The gut microbiome's role in AD pathogenesis likely stems from the gut–brain axis, a bi‐directional communication pathway between the enteric and central nervous systems. Dysbiosis seen in the gut can also lead to increased expression of amyloid precursor protein in mice, which can increase an individual's risk for neuroinflammation (Chalazonitis & Rao, 2018). One prior study found that patients with AD had a higher abundance of gut Bacteroidetes and decreased abundance of Actinobacteria (Vogt et al., 2017). Similar findings have been reported in mouse models of AD (Harach et al., 2017).

Oral microbiome dysbiosis has been previously associated with oral diseases, including dental caries and periodontitis. Additionally, experimental evidence suggests a link between oral bacteria and AD. Circulating levels of tumor necrosis factor‐alpha (TNF‐α) and antibodies for oral bacteria including *A. actinomycetemcomitans*, *T. forsythia*, and *P. gingivalis* have been found to be higher in AD patients’ serum compared to controls (Kamer et al., 2009). Furthermore, serum IgG levels to common periodontal microbiota are associated with risk for developing incident AD (Noble et al., 2014). Taken together, this evidence suggests a potential link between the oral microbiome and AD.

The mechanism by which the oral microbiota impacts cognition is likely mediated by changes in the oral microenvironment that select for pathogens and facilitate transmission of bacteria outside of the mouth. Dysbiosis, particularly due to antibiotic exposure, reduces the abundance of the protective commensal bacteria and enriches for pathogens (Socransky & Haffajee, 2005). The simple acts of chewing and brushing can leak bacteria and inflammatory markers into systemic circulation, particularly in those with local inflammation due to periodontitis. With adequate immune response, the transient bacteremia is eliminated from circulation. However, since many older adults experience a decline of the immune system (also known as immunosenescence), they are more likely to be immunocompromised and, thus, may not be able to clear the bacteremia. It has been suggested that immunosenescence also favors the overgrowth of oral anaerobes, leading to a pro‐inflammatory response that weakens the blood brain barrier, allowing bacteria to spread to the brain (Shoemark & Allen, 2015). Once pathogens and inflammatory markers migrate to the brain and penetrate the blood–brain barrier, they can influence microglial activation, Aβ deposition, tau protein phosphorylation, and vascular changes that could all contribute to the pathology of AD (Aarabi, Thomalla, Heydecke, & Seedorf, 2018; Lewy et al., 2018; Uppoor, Lohi, & Nayak, 2013).

### 4.2 Volatile organic compounds (VOCs): Metabolites detected in exhaled breath

Volatile organic compounds (VOCs) comprise a chemically diverse group of organic compounds that arise by a variety of catabolism routes, but principally from amino and fatty acids (Lundstrom, Hummel, & Olsson, 2003). Their low molecular weight (~500 Daltons), low boiling points, and high vapor pressure under ambient conditions allow them to readily diffuse through the gas phase and within biological systems; they serve as signaling molecules (e.g., hormones) and scents detectable by humans (Rowan, 2011). Thousands of VOCs are excreted in each breath, which have proven useful for diagnosing a broad range of diseases, including diabetes (Galassetti et al., 2005; Novak et al., 2007; Phillips, Cataneo, Cheema, & Greenberg, 2004), gastrointestinal and liver diseases (Probert et al., 2009), different types of cancer (Hakim et al., 2012; Horvath, Lazar, Gyulai, Kollai, & Losonczy, 2009; Mazzone, 2008; Phillips et al., 2010), and infections (Chambers, Scott‐Thomas, & Epton, 2012; Phillips et al., 2006; Ren et al., 2017). This innovative approach is now being applied to neurodegenerative disease research. Though only in its infancy, changes in breath‐derived VOC profiles are evident in several disease states, such as multiple sclerosis (Broza et al., 2017), AD (Mazzatenta, Pokorski, Sartucci, Domenici, & Di Giulio, 2015), Parkinson's disease (Nakhleh et al., 2015), and discriminating between the latter two neurodegenerative diseases are emerging (Lau, Yu, Lee, Huh, & Lim, 2017; Tisch et al., 2013). Mazzatenta et al., (2015 noted that AD patients exhibited a significant difference in breath frequency (*p* = .002) and maximum breath peak frequency (*p* = .02) compared to healthy subjects, possibly due to increased neuronal death or damage. These changes in respiration, along with utilizing VOCs, may become useful in predicting and diagnosing AD.

### 4.3 Salivary lipids and proteins

Saliva, or oral fluid, has long been of interest as a substitute for blood and other body fluids for disease diagnosis and disease/drug monitoring. Saliva is readily accessible, can be obtained non‐invasively, and contains a large number of analytes transferred by serum (e.g., cytokines, antibodies, hormones, VOCs, and drugs (Broza, Mochalski, Ruzsanyi, Amann, & Haick, 2015). Current salivary proteomic and transcriptomic knowledge for biomarker discovery includes oral cancer (Lee, Garon, & Wong, 2009; Park et al., 2009) and periodontal diseases (Christodoulides et al., 2007; Giannobile et al., 2009). Potential salivary biomarkers associated with neurodegenerative diseases have been recently reviewed (Farah et al., 2018). Changes with salivary protein levels, like Aβ peptides (Aβ40 and Aβ42), tau and lactoferrin, and salivary acetylcholinesterase activity have been linked to AD.

Lipids present in body fluid carry a number of signatures from the cells/organs released. These molecules include information reflective of cellular membrane structure, function, energy storage/metabolism, and signaling. Saliva is enriched with neutral lipids such as cholesterol, cholesteryl esters, mono‐, di‐ and triglycerides, and free fatty acids (Larsson, Olivecrona, & Ericson, 1996). A previous study of serum and saliva found moderate correlation of total cholesterol and triglycerides, indicating that serum lipoproteins contribute to the salivary lipids a great deal (Singh et al., 2014). However, in a preliminary study using a shotgun lipidomics platform (Han, Yang, & Gross, 2012; Wang, Wang, Han, & Han, 2016; Yang, Cheng, Gross, & Han, 2009), we quantified hundreds of polar lipid species, including phospholipids and sphingolipids (unpublished data) from saliva samples of a group of healthy individuals. We found that the profiles between plasma and salivary lipids were different. This observation indicates that salivary glands and other sources contribute a large amount of lipids to saliva and suggests that salivary lipids could be used for development of biomarkers beyond those of plasma.

Studies evaluating salivary lipids as markers for oral health or cognitive decline are limited. A previous study using lipid analysis of parotid saliva among two groups of female subjects susceptible to and resistant to dental caries showed higher total lipid concentration in the caries‐susceptible group (Tomita, Miyake, & Yamanaka, 2008), suggesting that salivary lipid levels play a role in caries development. We believe that changes in saliva lipids that occur in the context of neurodegeneration and/or declining cognition could be detected by lipidomics analysis and can serve as early biomarkers for neurological diseases including AD; however, further research is needed in this area.

## 5 CONCLUSIONS

Despite decades of research and promising preclinical studies, disease‐modifying treatments for AD remain elusive. Identifying AD‐susceptible individuals in early stages may be key for successfully developing disease‐modifying treatments. Numerous experimental and clinical studies suggest a link between oral health and the development of AD, suggesting that the oral cavity could be a source of important biomarkers of AD, as well as a potential modifiable target for AD prevention. Further research is needed to fully understand the value of oral biomarkers as predictors or mediators of AD development and progression.

## CONFLICT OF INTEREST

The authors of this study have no conflicts of interest relevant to this study to disclose.

## AUTHOR CONTRIBUTIONS

M.E.O., K.R.R., C.Y., E.H.Y., and X.H. conceived and designed the study. M.E.O., K.R.R., C.Y., E.H.Y., and X.H. drafted a significant portion of the manuscript or figures.
